# Clinical and Laboratory Characteristics of Individuals Aged ≤17 Years With Homeostatic Iron Regulator (HFE) p.C282Y Homozygosity, a Common Hemochromatosis Genotype

**DOI:** 10.7759/cureus.50043

**Published:** 2023-12-06

**Authors:** James C Barton, Jackson C Barton, Ronald T Acton

**Affiliations:** 1 Department of Medicine, University of Alabama at Birmingham, Birmingham, USA; 2 Southern Iron Disorders Center, Brookwood Baptist Medical Center, Birmingham, USA; 3 Department of Microbiology, University of Alabama at Birmingham, Birmingham, USA

**Keywords:** transferrin saturation, serum ferritin, phlebotomy, iron overload, iron, hfe p.c282y, hemochromatosis, children

## Abstract

Background

Characteristics of cohorts of individuals aged ≤17 years with homeostatic iron regulator (*HFE*) p.C282Y (rs1800562) homozygosity, a common hemochromatosis genotype, have not been reported.

Methodology

We retrospectively tabulated characteristics of white individuals aged ≤17 years with p.C282Y homozygosity. Individuals were not recruited for this study. We defined transferrin saturation (TS) >45%, serum ferritin (SF) >300 µg/L (M) and >200 µg/L (F) as elevated and liver iron grade 3 or 4, hepatic iron index >1.9 µmol Fe/g dry weight liver/y, and phlebotomy-mobilized iron >1.0 g (M) and >0.3 g (F) as increased.

Results

There were nine males and six females with a mean age of 12 ± 4 years (range = 5-17 years). The mean age of 10 probands (13 ± 3 years) was greater than that of five individuals discovered in family studies (9 ± 4 years) (p = 0.0403). Presenting manifestations of probands included fatigue/lethargy (5), elevated TS (2), and polycystic ovary syndrome, amenorrhea, and diabetes (2). In 15 individuals, the mean TS was 65 ± 23%. TS was elevated in 11 (73.3%) individuals aged 5-17 years. In 14 individuals, the mean SF was 262 ± 289 µg/L. SF was elevated and liver and phlebotomy-mobilized iron were increased in two male and three female probands aged 13-16 years (5/14 individuals, 35.7%). No individual had advanced hepatic fibrosis, arthropathy, hypogonadism, cardiomyopathy, or hyperpigmentation.

Conclusions

We conclude that five individuals aged 13-16 years (5/14 individuals, 35.7%) had increased liver and phlebotomy-mobilized iron.

## Introduction

Hemochromatosis is a group of heritable disorders that increase risks of developing iron overload by reducing hepcidin, the main controller of iron absorption, or by causing hepcidin resistance of ferroportin 1, the only iron exporter and hepcidin receptor [1]. Hemochromatosis in whites of Western European descent is usually due to homozygosity for a common missense allele p.C282Y (rs1800562) of the homeostatic iron regulator (*HFE*, chromosome 6p22.2) [2]. HFE, a non-classical class I major histocompatibility complex protein [2], is an upstream regulator of hepcidin and thus of iron homeostasis [1]. The estimated prevalence of p.C282Y homozygosity in US whites is 0.44% (95% confidence interval = 0.42-0.47) [3].

Clinical manifestations of adults with *HFE *p.C282Y homozygosity at presentation include weakness, lethargy, and abdominal pain [4]. Laboratory characteristics include elevated transferrin saturation (TS) and serum ferritin (SF) and increased liver and phlebotomy-mobilized iron [4]. Severe iron overload occurs predominantly in men [4]. Some adults with p.C282Y homozygosity also have advanced hepatic fibrosis, arthropathy, diabetes mellitus, hypogonadism, cardiomyopathy, or skin hyperpigmentation [4]. Non-*HFE *heritable and environmental variables modify iron loading in adults with hemochromatosis and p.C282Y homozygosity [4,5].

This retrospective study aimed to tabulate and describe characteristics of 11 reported [6-12] and four previously unreported individuals aged ≤17 years with *HFE *p.C282Y homozygosity. We discuss the characteristics of these 15 individuals and those of adults with p.C282Y homozygosity.

## Materials and methods

Subject identification

Each author performed independent computerized searches of medical literature published between August 1, 1996 [2] and July 30, 2023, using the following terms: adolescent, child, early age of onset, hemochromatosis, *HFE*, iron overload, juvenile, newborn, p.C282Y, TS, screening, and SF. Two authors (JCB and JCB) searched computerized medical records and charts at this center for the interval 1990-2020.

Subjects included

We included all individuals ascertained to have *HFE *p.C282Y homozygosity at ages ≤17 years who were reported (or the authors inferred) to be white residents of Western European or derivative countries. We did not recruit participants for this study. We defined probands as the first individuals in respective kinships diagnosed to have p.C282Y homozygosity. Other individuals were discovered in family studies.

Subjects excluded

We excluded individuals with *HFE *p.C282Y homozygosity who were also reported to have a deleterious mutation in another hemochromatosis-related gene (*HAMP*, *HJV*, *SLC40A1*, or* TFR2*). We also excluded individuals whose data were insufficient.

Clinical and laboratory characteristics

*HFE *p.C282Y homozygosity in 11 individuals was previously reported [6-12] and was ascertained in four previously unreported individuals evaluated at this center, as described elsewhere [13]. We defined TS >45% and SF >300 µg/L (M) and >200 µg/L (F) as elevated [3]. We defined increased liver iron as (1) histochemical iron grade 3 or 4; (2) increased measures using atomic absorption spectrometry or magnetic resonance; or (3) hepatic iron index >1.9 µmol Fe/g dry weight of liver/y [4]. Advanced hepatic fibrosis (F3 or F4) was defined by the interpretation of liver specimens by microscopy [14] or by clinical and imaging criteria consistent with cirrhosis. We also tabulated reports of the following conditions: arthropathy, diabetes, hypogonadism, cardiomyopathy, and skin hyperpigmentation. We defined increased phlebotomy-mobilized iron as >1.0 g (M) and >0.3 g (F) [15].

Statistics

Data consisted of observations of 15 individuals, although all observations were not available for each individual. Data for age, TS, and SF are displayed to the nearest integer. Kolmogorov-Smirnov testing demonstrated that age, TS, and SF data did not differ significantly from those that are normally distributed. Thus, we displayed these data as means ± 1 standard deviation and compared means using Student’s t-test for unpaired samples (two-tailed).

## Results

Characteristics of individuals aged ≤17 years with *HFE *p.C282Y homozygosity

These data are displayed in Table 1. There were nine males and six females with a mean age of 12 ± 4 years (range = 5-17 years). The mean age of 10 probands (13 ± 3 years) was greater than that of five individuals discovered in family studies (9 ± 4 years) (p = 0.0403). Countries represented by 15 individuals were Australia, England, France, Germany, Portugal, and the United States.

**Table 1:** Characteristics of individuals aged ≤17 years with HFE p.C282Y homozygosity. ^a^: Estimate based on the report of 10 units of blood removed by phlebotomy. ^b^: Liver biopsy and subsequent phlebotomy performed at age 17 years. ^c^: No *HAMP*, *HJV*, *SLC40A1*, or *TFR2 *mutations were detected in this individuals. Similar testing in other individuals was not reported. ^d^: *HFE *mutation analysis in this individual was performed by the referring physician, although the rationale for this analysis was/is unknown. ADHD: attention-deficit/hyperactivity disorder; ALT: serum alanine aminotransferase; AST: serum aspartate aminotransferase; HII: hepatic iron index, expressed as µmol Fe/g dry weight of liver/y; n.a.: not available/not applicable; PCOS: polycystic ovary syndrome; SF: serum ferritin; TS: transferrin saturation

Age, sex	Presentation	TS, %	SF, µg/L	Liver data	Phlebotomy-mobilized iron, g	Reference
5 M	Family study	94	41	n.a.	n.a	[12]
7 M	Family study	39	190	n.a.	n.a.	[9]
8 F	Bloody stools, abdominal pain, weight loss, iron deficiency	77	10	n.a.	n.a.	[6]
9 M	Family study	24	n.a.	n.a.	n.a.	[7]
9 M	Elevated TS	70	79	n.a.	n.a	[12]
11 M	Family study	61	93	n.a.	n.a	[12]
13 F	Fatigue, seizure disorder, ADHD	88	267	Normal ALT/AST, HII 5.0, early fibrosis	2.0^a^	[7]
13 F	Fatigue, male pattern hair loss, hirsutism, hypertestosteronemia, amenorrhea, diabetes mellitus, PCOS, pectus excavatum	70	900	Elevated ALT/AST, iron grade 3-4, HII 3.3, moderate steatosis^c^	3.5^b^	[11], present study
14 M^c^	Lethargy	51	572	Normal ALT, HII 4.2	2.0	[10]
14 F	Elevated TS	77	109	Normal liver function tests, HII 2.0, no fibrosis	n.a.	[8]
15 M	Fatigue	97	562	Elevated ALT/AST, iron grade 3-4, no fibrosis	1.6	Present study
15 M^d^	Fatigue, weakness, gastrocnemius equinus, ADHD	30	43	Normal ALT/AST	n.a	Present study
15 F	Family study	77	32	Normal ALT/AST	n.a	Present study
16 F	Male pattern hair loss, hirsutism, hypertestosteronemia, amenorrhea, diabetes mellitus, PCOS, symbrachydactyly	45	733	Elevated ALT/AST, iron grade 3-4, no fibrosis	3.0	[11], present study
17 M	Dizziness	82	40	Normal ALT/AST	n.a	Present study

Manifestations of probands at presentation included fatigue/lethargy (5), elevated TS (2), polycystic ovary syndrome (PCOS), amenorrhea, and diabetes (2), attention-deficit/hyperactivity disorder (ADHD) (2), and bloody stools, abdominal pain, and iron deficiency without anemia (1).

In 15 individuals, the mean TS was 65 ± 23%. Mean TS in probands (69 ± 21 µg/L) and in individuals discovered in family studies (59 ± 28 µg/L) did not differ significantly (p = 0.4603). TS was elevated according to the present criteria in 11 (73.3%) individuals aged 5-17 years. TS was elevated according to the CALIPER cohort reference ranges [16] in 10 (66.7%) individuals aged 5-17 years. In 14 individuals, the mean SF was 262 ± 289 µg/L. The difference between the mean SF in probands (332 ± 331 µg/L) and in individuals discovered in family studies (89 ± 73 µg/L) was not statistically significant. SF was elevated according to the present criteria in five probands aged 13-16 years (5/14 individuals, 35.7%). SF was elevated according to reference ranges established in German children and adolescents [17] in nine of the 15 (60.0%) individuals aged 7-16 years. Four probands (4/15 individuals, 26.7%) had both elevated TS and elevated SF according to the present criteria.

Six probands for whom data were available had an elevated hepatic iron index and/or grade 3-4 liver iron. One of these had early hepatic fibrosis without non-iron liver disease and another had moderate hepatic steatosis. Two females with PCOS had diabetes. There was no report of advanced hepatic fibrosis, arthropathy, hypogonadism, cardiomyopathy, or skin hyperpigmentation. Five probands (two males, three females) had increased phlebotomy-mobilized iron.

## Discussion

Novel aspects of this case series are tabulation and description of characteristics of 15 individuals aged ≤17 years with *HFE *p.C282Y homozygosity. We infer that *HFE *genotyping was performed in these individuals because they had elevated TS or elevated SF (n = 9), because p.C282Y homozygosity had been diagnosed in other family members (n = 5), and for an inapparent reason(s) (n = 1). Ten subjects were probands. Likewise, adult probands with p.C282Y homozygosity often have family members with p.C282Y homozygosity [18]. The present results suggest that p.C282Y homozygosity should be considered in the differential diagnosis of elevated TS in asymptomatic children. Five of the present probands (50.0%) reported fatigue/lethargy. In another study, weakness or lethargy was reported by 54.2% of 59 Utah adult hemochromatosis probands [4]. In the present individuals, the most common laboratory abnormalities were elevated TS and SF and increased liver and phlebotomy-mobilized iron, consistent with cohorts of adults with p.C282Y homozygosity [4].

TS, SF, or both were elevated in 73.3%, 35.7%, and 26.7% of the present individuals, respectively. Thus, elevated TS was the most common iron-related abnormality. In 82 white men discovered to have *HFE *p.C282Y homozygosity in a primary care-based screening program, TS, SF, or both were elevated in 82.9%, 87.8%, and 76.8%, respectively [19]. In 131 white women discovered to have p.C282Y homozygosity in the same screening program, TS, SF, or both were elevated in 74.8%, 57.3%, and 48.9%, respectively [19]. These observations substantiate that TS and SF phenotypes in p.C282Y homozygotes are variable [4,20] and that the occurrence of p.C282Y homozygosity cannot be excluded in individuals whose TS or SF values are not elevated, regardless of age.

In five of the present probands aged 13-16 years, the mean SF was 607 ± 235 µg/L, and the mean phlebotomy-mobilized iron was 2.4 ± 0.8 g. In 160 adults aged 42 ± 1 years with *HFE *p.C282Y homozygosity without advanced hepatic fibrosis, the mean SF was 1,181 ± 74 µg/L, and the mean phlebotomy-mobilized iron was 5.0 ± 0.3 g [14]. Together, these observations substantiate that excess iron accumulation occurs in some p.C282Y homozygotes aged ≤17 years, although their iron accumulation is less severe at this age than that which is typical of adults with p.C282Y homozygosity.

PCOS occurs in ~5.4% of non-Hispanic white females aged 16-40 years in the United States [21]. Two females with PCOS in the present study had amenorrhea, diabetes, elevated SF, and increased liver and phlebotomy-mobilized iron [11]. Females with PCOS have lower plasma hepcidin levels [22] and higher testosterone levels [23] than healthy females without PCOS, although testosterone levels in females with PCOS are lower than those of age-matched males [23]. Testosterone is associated with suppressed hepcidin in men [24]. Together, amenorrhea and lower hepcidin levels may increase the risk of excess iron accumulation in females with PCOS and *HFE* p.C282Y homozygosity.

One of the present probands with PCOS and moderate hepatic steatosis also had elevated SF, diabetes, and increased phlebotomy-mobilized iron. In adults with *HFE *p.C282Y homozygosity, non-alcoholic hepatic steatosis was associated with higher SF and greater diabetes prevalence but not greater phlebotomy-mobilized iron [25].

ADHD (or a learning disability) was diagnosed in 14.7% of non-Hispanic US whites aged 3-17 years during the interval 2016-2018 [26]. Two of the present probands (13.3%) were also diagnosed to have ADHD. Thus, the prevalence of ADHD observed among the probands in this study was similar to that reported for non-Hispanic US whites aged 3-17 years (24).

A strength of the present retrospective study is an analysis of available reports of *HFE *p.C282Y homozygosity and iron phenotypes in individuals aged ≤17 years. Prospective studies have reported prevalence estimates of p.C282Y homozygosity without iron phenotyping in newborns [9]. In clinical settings, *HFE *mutation analysis in children is not recommended unless they have iron phenotypes suggestive of hemochromatosis or iron overload [27,28].

Limitations of this work include the following: (1) the available sample size was small; (2) we may have overlooked reports of other individuals aged ≤17 years with *HFE *p.C282Y homozygosity; (3) we included only white subjects; and (4) there were insufficient numbers of subjects to permit comparisons of clinical and laboratory characteristics of males and females of similar age. Another limitation is that evaluations for deleterious mutations in *HAMP*, *HJV*, *SLC40A1*, or *TFR2 *were not reported in 14 (93.3%) of the present individuals. It is plausible, although unproven, that the occurrence of *HFE *p.C282Y homozygosity and heterozygosity or homozygosity for a deleterious *HAMP*, *HJV*, *SLC40A1*, or *TFR2 *allele(s) would have been associated with more severe iron phenotype(s) than were reported in the present individuals.

Uncertainties of this work include whether the present individuals are representative of larger cohorts of individuals aged ≤17 years diagnosed to have *HFE *p.C282Y homozygosity or discovered to have p.C282Y homozygosity in family studies or population-based screening.

## Conclusions

The present analyses of characteristics of hemochromatosis and *HFE *p.C282Y homozygosity in 15 individuals aged ≤17 years reveal features that heretofore have been extrapolated from studies of adults, were suggested by investigations of individual kinships, or were unknown. The most common subjective complaint in 10 probands was fatigue/lethargy. In 73.3% of individuals, either TS, SF, or both were elevated. Five individuals had increased liver and phlebotomy-mobilized iron, although there were no reports of advanced hepatic fibrosis, arthropathy, hypogonadism, heart disease, or skin hyperpigmentation.
